# The mechano-immunological landscape in the tumor microenvironment: From mechanical sensing to a new therapeutic paradigm

**DOI:** 10.1016/j.mtbio.2026.103317

**Published:** 2026-06-04

**Authors:** Wen Li, Yuan-Yuan Xin, Ming-Zhu Jin, Wei-Lin Jin

**Affiliations:** a Institute of Cancer Neuroscience, Medical Frontier Innovation Research Center, The First Hospital of Lanzhou University, The First Clinical Medical College of Lanzhou University, Lanzhou, 730000, PR China; b Department of Obstetrics and Gynecology, Renji Hospital, Shanghai Jiao Tong University School of Medicine, Shanghai, 200001, PR China; c Shanghai Key Laboratory of Gynecologic Oncology, Renji Hospital, Shanghai Jiao Tong University School of Medicine, Shanghai, 200001, PR China

**Keywords:** Mechano-immunological landscape, Mechanotransduction, Mechano-immunotherapy, Immune checkpoint, Therapeutic paradigm, PIEZO1

## Abstract

The tumor microenvironment (TME) is a complex ecosystem where mechanical forces are now recognized not as passive byproducts but as active drivers critically shaping anti-tumor immunity. This review introduces the integrative concept of the “Mechano-immunological Landscape” (MIL) to delineate the dynamic and spatially heterogeneous network of interactions between mechanical cues and immune cells within tumors. We systematically elaborate on its three core pillars: Mechano-immunological Checkpoints, Mechano-immune Memory, and Landscape Plasticity. Building upon this framework, we propose a novel therapeutic paradigm termed “Mechano-immunological Landscape Remodeling Therapy” (MILRT). This paradigm discusses potential strategies to reverse immune suppression and enhance anti-tumor efficacy by modulating the mechanical TME, including matrix de-stiffening, mechanical empowerment of immune cells, and targeting mechano-immunological checkpoints. Finally, we outline future research trajectories, pivotal challenges, and clinical translation prospects in this burgeoning field. We position MILRT as a multi-pronged and synergistic framework that leverages advanced biomaterials and biophysical tools to overcome current limitations in drug delivery and immunotherapy resistance, thereby paving the way for next-generation, mechano-aware immunotherapies and redefining the future of solid tumor treatment.

## Highlights

• Introduces the novel “Mechano-immunological Landscape (MIL)” concept as an integrative framework for decoding tumor mechanics—immune interplay.
• Systematically elaborates the three MIL pillars: mechano-immunological checkpoints, mechano-immune memory, and landscape plasticity.
• Proposes a transformative “MIL Remodeling Therapy (MILRT)” paradigm, combining matrix de-stiffening, immune cell empowerment, checkpoint blockade, and physical adjuvants.
• Identifies mechano-sensors (e.g., PIEZO1, YAP/TAZ) as actionable druggable targets within the immunosuppressive tumor microenvironment.
• Outlines a translational roadmap integrating mechano-immunophenotyping, artificial intelligence, and smart biomaterials for personalized therapy.

## Graphical abstract

The Mechano-immunological Landscape (MIL) and its therapeutic remodeling in the tumor microenvironment.The MIL integrates three core pillars—Mechano-immunological Checkpoints (e.g., Piezo1, YAP/TAZ), Mechano-immune Memory, and Landscape Plasticity—to form a dynamic framework of physical immunosuppression. Mechano-immunological Landscape Remodeling Therapy (MILRT) employs four synergistic strategies—Matrix De-stiffening, Mechanical Empowerment of Immune Cells, Checkpoint Blockade, and Physical Adjuvant Therapies—to convert immune-cold tumors into immune-hot. Created with BioRender.

**Figure:**
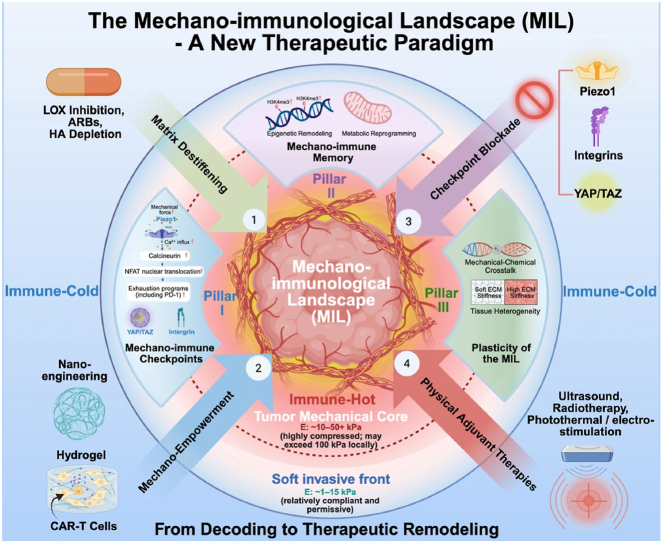

## 1 Introduction

The research paradigm of the tumor microenvironment (TME) is undergoing a profound shift from a “biochemical-centric” view to a “mechano-biochemical synergy” perspective. While longstanding focus has been on biochemical signaling networks such as growth factors, cytokines and chemokines, the TME is increasingly recognized as a highly structured physical entity [1,2]. This entity is characterized by abnormal stiffening of the extracellular matrix (ECM), continuous accumulation of solid stress, and significantly elevated interstitial fluid pressure [[3], [4], [5]]. These mechanical properties are not merely passive consequences of tumor growth; rather, they are key factors that actively participate in driving malignant progression by activating specific mechanotransduction pathways [[6], [7], [8]]. Our previous review systematically described the TME as a fundamental component and therapeutic target of a complex ecosystem, emphasizing the potential of drug repurposing in this field [9]. Building upon the original “biochemical landscape”, it has become crucial to incorporate a comprehensive understanding of the mechanical dimension of the TME into current research frameworks [10].

The deep integration of mechanobiology and immunology has given rise to the emerging interdisciplinary field of “Mechanoimmunology”, redefining our understanding of the interaction between the immune system and the mechanical microenvironment [11]. This field reveals that immune cells are equipped with sophisticated “mechanical sensors”, including mechanosensitive ion channels (such as PIEZO1) [12], integrin adhesion molecules [13], and mechanosensitive transcription factors (such as YAP/TAZ) [14]. These sensors enable immune cells to sense and respond to physical signals within the TME, thereby dynamically regulating their activation, differentiation, migration, and effector functions [12]. Studies have shown that dysregulated mechanical sensing is an important mechanism underlying tumor immune escape [15]. For instance, matrix stiffness can suppress T-cell cytotoxicity through PIEZO1-mediated calcium influx and direct CD8^+^ T cells toward a state of functional exhaustion [16,17]. Similarly, in macrophages, mechanical signals drive polarization towards the immunosuppressive M2 phenotype through the YAP/TAZ pathway, while also reshaping the metabolic program to enhance aerobic glycolysis and support tumor-promoting functions [18,19]. These findings establish mechanical microenvironment as a new dimension of immune regulation, providing a mechanistic explanation for the poor responsiveness of physically barrier-rich “immune-cold tumors” to immunotherapy [20,21].

**Fig. 1:**
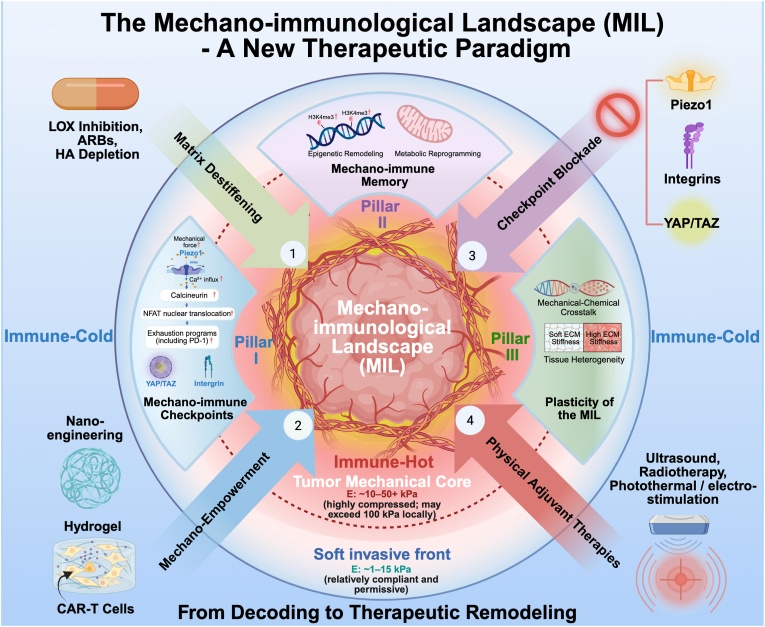
**The Mechano-immunological Landscape (MIL): From Conceptual Framework to Therapeutic Remodeling.** This schematic diagram illustrates the core concepts and therapeutic strategies of the Mechano-immunological Landscape (MIL) within the tumor microenvironment (TME), with quantitative and mechanistic details. The MIL is depicted as a dynamic, multi-layered system comprising three core pillars: (1) Mechano-immunological Checkpoints (e.g., Piezo1, Integrins, YAP/TAZ), which sense and transduce physical signals (e.g., Piezo1-Ca^2+^ signaling directly upregulates PD-1 expression via NFAT nuclear translocation); (2) Mechano-immune Memory, underpinned by epigenetic remodeling (e.g., H3K27me3) and metabolic reprogramming; and (3) Landscape Plasticity, driven by mechanical heterogeneity (e.g., tumor core stiffness: 10–50 kPa, invasive front: 1–15 kPa) and mechanical-chemical crosstalk. Emerging from this framework is the novel therapeutic paradigm Mechano-immunological Landscape Remodeling Therapy (MILRT), which employs four synergistic strategies to convert “immune-cold” tumors into “immune-hot”: (1) Matrix De-stiffening (e.g., LOX inhibition, ARBs), (2) Mechanical Empowerment of Immune Cells (e.g., nanomaterial/hydrogel empowerment of CAR-T cells), (3) Blockade of Mechano-immunological Checkpoints, and (4) Physical Adjuvant Therapies (e.g., ultrasound, radiotherapy). Together, this integrative approach aims to overcome physical immunosuppression and redefine the future of solid tumor immunotherapy. MIL, Mechano-immunological Landscape; LOX, Lysyl Oxidase; ARBs, Angiotensin Receptor Blockers; HA, Hyaluronic Acid; CAR-T, Chimeric Antigen Receptor T cell; H3K4me3, Histone H3 Lysine 4 Trimethylation; Piezo1, Piezo-type mechanosensitive ion channel component 1; YAP, Yes-associated protein; TAZ, Transcriptional coactivator with PDZ-binding motif; ECM, Extracellular Matrix. This figure was created using BioRender (https://biorender.com/).

Although several independent mechanical-immune interaction axes have been described, the field still lacks an integrated concept to capture this complex and dynamics of this network within the TME. Therefore, we propose the core concept of the “Mechano-immunological Landscape” (MIL). The MIL aims to systematically depict the functional sub-networks—characterized by spatial heterogeneity and temporal dynamics—formed by the interplay between the mechanical properties of tumors and immune cells behavior [22]. Its core consists of “three major pillars”: mechanical immune checkpoints (e.g., PIEZO1, integrins, YAP/TAZ) [23], mechanical immune memory [24], and landscape plasticity [25]. This review will first elaborate on the three pillars of the MIL and then detail the MILRT paradigm and its future prospects. Crucially, the translation of MILRT from concept to clinical reality is inherently linked to advancements in bioengineering and biomaterials science—ranging from the design of smart matrices for immune cell training to the development of targeted nano-therapeutics and responsive physical stimulation devices (Fig. 1). This synergy underscores the pivotal role of biomaterial-based strategies and advanced drug delivery systems in deciphering and reprogramming the physical language of tumor immunity, a core interest of pharmacological science.

## 2 The core pillars of the mechano-immunological landscape

The establishment and maintenance of the Mechano-immune Landscape(MIL) rely on three core pillars: Mechano-immune Checkpoints [23], Mechano-immune Memory [24], and the plasticity of the Mechano-immune Landscape [25]. These three pillars form a dynamic and multi-level interaction network that converts mechanical signals within the TME into the core driving force for immune regulation, profoundly influencing the fate, function and anti-tumor efficacy of immune cells [[26], [27], [28]]. Understanding these pillars not only reveals the physical basis of tumor immune evasion but also provides theoretical support for mechano-immunological intervention strategies.

### 2.1 Pillar I: mechano-immunological checkpoints

Traditional immune checkpoints (such as PD-1, CTLA-4) are largely limited to protein-protein interactions [[29], [30], [31]]. However, within the complex tumor mechanical environment, immune regulation has evolved beyond molecular recognition into the realm of “mechano-immunological checkpoints"—pathways capable of sensing, transducing, and integrating physical stimuli [32,33]. These pathways convert external mechanical signals (e.g., stretch, shear force, and matrix stiffness) into immune regulatory signals [[34], [35], [36]], thereby reshaping the immune landscape at the molecular and cellular levels by regulating the immune cell activation, differentiation, metabolism, and effector functions [37,38].

#### 2.1.1 Ion channel checkpoints: the role of PIEZO1 in T cells, macrophages, and innate immunity

PIEZO1, as a key mechanosensitive cation channel, acts as a central mechanosensor in various immune cells [39,40]. It mediates calcium ion (Ca^2+^) influx by sensing mechanical stimuli such as extracellular matrix (ECM) stiffness and fluid shear stress [41], thereby triggering downstream signaling pathways and regulating immune cell functional states [42].

In T cells, PIEZO1 senses tumor matrix stiffness signals [43]. In a high-stiffness environment (e.g., >20 kPa), chronic PIEZO1 activation leads to sustained Ca^2+^ influx, which can induce metabolic stress, mitochondrial dysfunction, and alter calcineurin-NFAT signaling dynamics [44,45]. This cascade inhibits T cell cytotoxicity and promotes CD8^+^ T cell differentiation into a functionally exhausted state [16,46]. Specifically, prolonged PIEZO1-mediated calcium signaling may dysregulate the precise spatiotemporal control required for effective NFAT activation, ultimately reducing production of effector molecules like IFN-γ and TNF-α by up to 60% while upregulating exhaustion markers such as PD-1 and TIM-3 by 3- to 4-fold [47]. Conversely, inhibiting PIEZO1 can enhance T cell metabolic adaptability and effector persistence, suggesting its role as a targetable “plasticity node” in immunotherapy [16].

In macrophages, PIEZO1 activation promotes polarization towards a tumor-promoting M2 phenotype through a Ca^2+^-dependent AMPK/mTOR pathway and significantly enhances aerobic glycolysis, providing metabolic support for tumor growth [48]. Quantitative studies have shown that on stiff substrates (25–50 kPa), M2 marker expression (CD206, Arg1) increases by approximately 2.5-fold compared to soft substrates (2–5 kPa), while M1 markers (iNOS, TNF-α) decrease by 40% [18,48]. PIEZO1 also plays a crucial role in innate immunity. Dendritic cells (DCs) sense matrix stiffness through PIEZO1 to regulate their metabolic reprogramming (e.g., shift from mitochondrial respiration to glycolysis) and antigen-presenting capacity [49]. Specifically, DCs cultured on stiff matrices (50 kPa) exhibit a 2-fold increase in glycolysis rate and a 1.8-fold increase in antigen cross-presentation ability relative to soft matrices (5 kPa) [49]. PIEZO1 is also a key sensor of periodic fluid shear stress, which is crucial for host defense [33]. In Natural Killer (NK) cells, PIEZO1 activation similarly affects killing efficiency and the ability to invade 3D matrices, indicating its broad significance in innate immune cytotoxicity [50]. Recent data indicate that NK cells exposed to fluid shear stress of 1–2 dyn/cm^2^ exhibit a ∼50% reduction in killing efficiency compared to static conditions, while inhibition of PIEZO1 restores cytotoxicity by 70% [50,51]. Furthermore, NK cell invasion into 3D matrices with stiffness of 10–20 kPa is enhanced by 2-fold relative to 1 kPa matrices [52].Therefore, PIEZO1 functions as a critical node connecting mechanical input and metabolic output across both innate and adaptive immunity (Table 2).

**Table 1:** Strategies and mechanisms of targeted mechano-immunological landscape remodeling therapy (MILRT).

Strategy Category	Specific Approach	Mechanism and Effects	Representative Agents /Technologies/Methods	Reference
**Strategy I: Tumor Mechanical Microenvironment “De-stiffening”**	LOX/LOXL2 Inhibitors	Inhibit collagen cross-linking enzymes, reduce ECM stiffness, enhance T-cell infiltration, synergize with PD-1 blockade	LOX/LOXL2 inhibitors (e.g., Simtuzumab, preclinical and early translational studies)	[[53], [54], [55], [56]]
	Angiotensin Receptor Blockers (e.g., Losartan)	Attenuate TGF-β–driven fibrosis, reduce collagen synthesis, decrease tumor stiffness, improve perfusion, enhance drug and immune cell delivery	Losartan	[57,58]
	Hyaluronidase	Degrade tumor-associated HA, reduce IFP, improve vascular permeability, facilitate immune cell entry into TME	PEGPH20 (PEGylated recombinant human hyaluronidase, preclinical and early-phase studies)	[59,60]
**Strategy II: Immune Cell “Mechanical Empowerment”**	*In vitro* Mechanical Training	Expand CAR-T/NK cells in stiffness-tunable 3D hydrogels simulating tumor stiffness to enhance mechanical adaptability and functional persistence	Stiffness-tunable 3D hydrogel expansion systems	[61,62]
	Nanomaterial Regulation	Tune nanoparticle elasticity to activate PIEZO1 in macrophages, promoting M2 to M1 polarization, or serve as delivery vectors for immunomodulators	Elasticity-tunable nanoparticles	[63]
**Strategy III: Mechano-immunological Checkpoint Blockade**	Modulating PIEZO1	Regulate PIEZO1 activity to prevent Ca^2+^-mediated T-cell exhaustion in stiff TME and restore cytotoxicity	Yoda1 (agonist), GsMTx4 (inhibitor)	[64,65]
	YAP/TAZ-TEAD Inhibitors	Inhibit YAP/TAZ transcriptional activity, block mechano-driven immune suppression and tumor progression	Small-molecule preclinical YAP/TAZ-TEAD interaction inhibitors	[66,67]
	Integrin Function Modulation	Modulate integrin function (e.g., β2) via agonistic or blocking antibodies to enhance T-cell infiltration or preserve stem-like memory phenotypes	Integrin-targeting antibodies or modulators	[68,69]
**Strategy IV: Physical Therapies as Immune Adjuvants**	Ultrasound Therapy	Induce immunogenic cell death via acoustic radiation force and cavitation, improve vascular function, reduce IFP, promote immune cell infiltration	Low-intensity pulsed ultrasound/Focused ultrasound	[70]
	Radiotherapy	Damage ECM, reduce local stiffness, release tumor neoantigens, reshape local immune landscape, convert “cold” to “hot” tumors	Radiotherapy combined with immune checkpoint inhibitors	[71,72]
	Photothermal/Electro-stimulation	Induce immunogenic cell death via thermal stress or modulate cell membrane potential and ion channels to influence immune cell mechanosensing and migration.	Photothermal therapy, electrical stimulation devices	[[73], [74], [75]]

**Table 2:** Quantitative effects of PIEZO1-Mediated mechanical signaling in innate immune cells.

Immune Cell Type	Mechanical Cue/Condition	PIEZO1-Related Mechanism	Quantitative Functional Changes	Biological Outcome	Reference
Macrophages	Stiff substrates (25–50 kPa) vs. soft substrates (2–5 kPa)	PIEZO1-mediated Ca^2+^ influx activates the AMPK/mTOR and YAP pathways, promoting glycolytic reprogramming	M2 markers (CD206, Arg1) ↑ ∼2.5-fold; M1 markers (iNOS, TNF-α) ↓ ∼40%	Enhanced M2 polarization and tumor-promoting immunosuppression	[18,48]
Dendritic Cells (DCs)	Stiff matrix (50 kPa) vs. soft matrix (5 kPa)	PIEZO1 regulates metabolic switching from oxidative phosphorylation to glycolysis and enhances antigen presentation	Glycolysis ↑ ∼2-fold; antigen cross-presentation ↑ ∼1.8-fold	Altered DC activation and T-cell priming capacity	[49]
Natural Killer (NK) Cells	Fluid shear stress (1–2 dyn/cm^2^)	PIEZO1-mediated mechanosensing regulates cytoskeletal tension and cytotoxic signaling	Killing efficiency ↓ ∼50% under shear stress; PIEZO1 inhibition restores cytotoxicity by ∼70%	Reduced NK-mediated tumor killing under mechanical stress	[33,50,51]
	3D matrices with stiffness 10–20 kPa vs. 1 kPa	PIEZO1-dependent mechanoadaptation promotes migration and matrix invasion	NK-cell invasion into 3D matrices ↑ ∼2-fold	Enhanced infiltration capacity in mechanically permissive matrices	[52]

Table note: AMPK, AMP-activated protein kinase; mTOR, mechanistic target of rapamycin; DC, dendritic cell; NK, natural killer; Arg1, arginase-1; iNOS, inducible nitric oxide synthase.

**Table 3:** Frontier therapeutic approaches for de-stiffening the tumor mechanical microenvironment.

Agent	Signal Target	Representative Cancer Type(s)	Study IDs	Development Phase
Simtuzumab(GS-6624)	LOX/LOXL2 inhibitors	PDAC	NCT01472198	Phase II
PXS-5505	LOX inhibitor	Myelofibrosis	NCT04644029	Phase I
Losartan	Unknown	PDAC	NCT01821729	Phase II
Losartan	Unknown	PDAC	NCT03563248	Phase III
PEGPH20	Unknown	HA-high metastatic PDAC	NCT01959139	Phase III
PEGPH20	Unknown	PDAC	NCT02715804	Phase Ib/II

Table note: PDAC, pancreatic ductal adenocarcinoma.

#### 2.1.2 Adhesion molecule checkpoints: mechanical signal transduction via integrins (e.g., β2 and αV)

Integrins serve as the core bridge connecting the extracellular mechanical environment with the intracellular actin cytoskeleton [76]. They function not only as mediators of cell adhesion but also as precise “mechanical sensors,” converting physical cues such as ECM stiffness and traction forces into intracellular biochemical signals [77]. This conversion fundamentally regulates the activation, migration, and effector functions of immune cells.

In T cells, the mechanical signal transduction function of β2 integrins (such as LFA-1) is particularly prominent [78]. Studies have shown that mechanical forces acting on β2 integrins can induce conformational changes, activate downstream focal adhesion kinase (FAK)/Pyk2 and Rho GTPase signals, and thereby decouple the proliferation and differentiation programs of T cells [79,80]. This decoupling effect is conducive to generating and maintaining memory T cells or CAR-T cells with stem cell-like characteristics, which are characterized by high expression of TCF-1, low expression of effector molecules, and stronger persistence and self-renewal ability [68]. This provides a theoretical basis for preparing “mechanically empowerment” cell therapy products through *in vitro* mechanical pre-conditioning (e.g., expansion within hydrogels of specific stiffness) [81].

Furthermore, mechanically activated integrins directly regulate immune synapse function [82]. In cytotoxic T cells and NK cells, integrin-mediated mechanical activation guides the polarized secretion of cytolytic granules (e.g., perforin, granzymes) toward the immune synapse, directly enhancing killing efficiency against target cells [83]. Thus, integrins are not merely adhesion molecules but also core checkpoints for mechanical sensing and effector output in immune cells.

#### 2.1.3 Transcriptional regulation checkpoints: YAP/TAZ as core amplifiers of mechanical signals

YAP (Yes-associated protein) and TAZ (Transcriptional coactivator with PDZ-binding motif) are core transcriptional co-activators downstream of the Hippo pathway, highly sensitive to cell tension, shape, and ECM stiffness [[84], [85], [86]]. As amplifiers of mechanical signals, they widely regulate gene expression in tumor cells and immune cells, and are key integration nodes in the mechanical immune landscape [87,88].

The YAP/TAZ-mediated immune cell polarization switch is collectively determined by mechanical stiffness thresholds, co-signaling molecules, and tumor type-specific differences. Regarding mechanical stiffness thresholds, it has been demonstrated that soft substrates (1–5 kPa) promote M2 polarization via nuclear YAP retention, while stiff substrates (>20 kPa) induce M1-like phenotypes with YAP/TAZ cytoplasmic sequestration and NF-κB activation, reporting a ∼3-fold increase in M2 marker CD206 on 2 kPa gels and a ∼4-fold increase in M1 marker iNOS on 50 kPa gels [19]. Furthermore, co-signaling molecules such as IL-4, IFN-γ, and TGF-β synergize with mechanical cues: studies have shown that IL-4 enhances YAP nuclear translocation on intermediate stiffness (∼10 kPa), while IFN-γ suppresses YAP activity on stiff matrices, shifting polarization toward M1; additionally, TGF-β activates YAP/TAZ to promote M2-like gene programs in soft microenvironments [89]. Tumor type-specific differences also play a critical role, as quantitative data highlight that in breast cancer, tumor-associated matrix stiffness (∼8–12 kPa) preferentially induces M2 polarization through YAP/TEAD transcriptional activity, whereas in pancreatic cancer, higher stiffness (∼25–35 kPa) combined with desmoplastic signals drives a mixed M1/M2 phenotype with a bias toward pro-inflammatory states [90]. Similarly, it has been reported that in lung cancer, 10–15 kPa matrices enhance M2 markers (Arg1, IL-10) by ∼5-fold via YAP binding to the Arg1 enhancer [91].

In immune cells, YAP/TAZ activity directly regulates their polarization and function, although the outcomes can be context-dependent. Some studies indicate that when macrophages are cultured on stiff substrates, increased matrix rigidity can enhance Piezo1-mediated mechanosensing and activate YAP signaling, promoting a pro-inflammatory M1 polarization in certain settings [18,89,92]. However, as elaborated in section 2.2.2, other evidence strongly supports that sustained mechanical signaling through YAP/TAZ predominantly drives and maintains the immunosuppressive M2 phenotype, highlighting the complexity of mechano-regulation [18,89,92].

In tumor cells, YAP/TAZ activation frequently induces immune checkpoint molecules such as PD-L1, thereby contributing to immune evasion through suppression of T cell activity [[93], [94], [95]].

Notably, the YAP/TAZ pathway engages in profound crosstalk with innate immune signaling. In stromal cells, YAP/TAZ activation can inhibit the cGAS-STING pathway, thereby modulating cell senescence and innate immune sensing [96]. Conversely, in tumor cells, ECM stiffening activates YAP/TAZ via mechanotransduction, which in turn impairs cGAS-mediated immune signaling, revealing a novel mechanically driven mechanism for evading immune surveillance [37]. Therefore, YAP/TAZ serves not only as a mechanical signal amplifier but also as a transcriptional hub for immune escape within the TME.

#### 2.1.4 Emerging checkpoints: mechano-immunological regulation by PYK2 and OSR2

As research progresses, additional molecules involved in mechano-immunological regulation continue to be identified. Among these, the kinase PYK2 and the transcription factor OSR2 are particularly notable [17,97,98].

PYK2, a non-receptor tyrosine kinase, acts as a mechano-immunological checkpoint in stiff and fibrotic TMEs such as pancreatic ductal adenocarcinoma (PDAC) and triple-negative breast cancer (TNBC) [99,100]. In PDAC, PYK2 integrates integrin-mediated mechanical signals with growth factor receptor pathways [101]. Biomechanical activation of PYK2 drives monocyte differentiation into immunosuppressive M2-like macrophages and simultaneously impairs CD8^+^ T cell motility in collagen-rich matrices [102,103]. Quantitative studies demonstrate that PYK2 phosphorylation increases 3-fold in tumors with stiffness >15 kPa compared to normal tissue [102]. Small-molecule inhibitors, such as PF-562271 and the more selective VS-4718, have shown preclinical efficacy: in a PDAC model, PF-562271 reduced M2 macrophage polarization by 60%, increased intratumoral CD8^+^ T cell density by 4-fold, and synergized with anti-PD-1 therapy to achieve complete regression in 30% of mice [102,104]. In TNBC, PYK2 inhibition reversed matrix-induced T cell suppression and improved the efficacy of adoptive T cell transfer [105]. A phase I clinical trial of PF-562271 in solid tumors (NCT00632112) demonstrated a manageable safety profile (diarrhea, fatigue), but efficacy was limited, partly due to off-target effects [106]. Next-generation inhibitors with improved selectivity (e.g., S-7) are under preclinical development. These data validate PYK2 as an actionable checkpoint, though clinical translation requires better isoform-selective agents.

OSR2 represents a novel class of mechano-immunological checkpoint that directly exacerbates CD8^+^ T cell exhaustion [17]. Mechanistically, mechanical stress (e.g., matrix stiffness >20 kPa) upregulates OSR2 expression in CD8^+^ T cells through the YAP/TEAD pathway [107]. OSR2 then recruits the histone methyltransferase G9a to silence effector-gene loci (e.g., Ifng, Prf1) while promoting expression of exhaustion-associated genes (e.g., Pdcd1, Havcr2), thereby driving epigenetic reprogramming toward terminal exhaustion [17,108]. In a murine melanoma model, OSR2-deficient CD8^+^ T cells showed 2.5-fold higher IFN-γ production and maintained polyfunctionality after chronic antigen exposure [17]. Pharmacological inhibition of OSR2 has not yet been achieved, but disruption of its interaction with G9a using a cell-permeable peptide restored T cell effector function by 70% *in vitro* [109]. Additionally, combining OSR2 knockdown with anti-PD-1 therapy in a stiff-matrix lung cancer model produced a 5-fold increase in complete response rate compared to anti-PD-1 alone [110]. These findings establish OSR2 as a central epigenetic hub linking mechanical stress to T cell exhaustion and highlight the therapeutic potential of targeting OSR2–G9a axis, although small-molecule inhibitors remain to be developed.

The discovery of these emerging checkpoints significantly expands the scope of mechano-immunological checkpoints and reveals the complex mechanisms through which mechanical signals finely tune immune responses via multi-level molecular nodes. Future efforts should focus on structure-based drug design for OSR2 and the development of highly selective PYK2 inhibitors to translate these targets into clinical mechano-immunotherapy.

### 2.2 Pillar II: mechano-immunological memory

Mechano-immune Memory refers to the phenomenon wherein immune cells, following short-term mechanical stimulation, undergo long-term changes in their phenotype, epigenetic state, and metabolic programming [111,112]. This “memory” can persistently affect immune cell function even after the initial mechanical cue is removed [113,114]. This concept links mechanical exposure to the long-term adaptability of immune cells and is a crucial manifestation of MIL dynamics.

#### 2.2.1 T-cell mechanical memory and stemness maintenance

T cells exhibit a striking form of mechanosensitive memory [84]. Research shows that T cells activated and expanded on stiff substrates maintain higher proliferative potential and effector function even after transfer to soft substrates or non-stimulatory environments [115]. This memory is underpinned by profound “epigenetic remodeling” and “metabolic reprogramming” [116].

Mechanistically, mechanical signals (e.g., via β2 integrin) can induce enrichment of specific histone modifications (e.g., H3K4me3, H3K27ac) at gene loci such as TCF7, maintaining an open chromatin state for genes that promote stem cell-like properties [[117], [118], [119]]. Concurrently, mechanical memory is accompanied by sustained metabolic reprogramming, such as enhanced oxidative phosphorylation and fatty acid oxidation, supporting long-term survival and self-renewal [68]. This form of memory, imprinted via stable epigenetic changes, has also been widely observed in cancer cells [24].

This discovery has significant translational value. Through *in vitro* mechanical preconditioning. For instance, “expanding CAR-T cells in 3D hydrogels mimicking the high stiffness of tumor tissues”, these cells can be “trained” in advance to acquire adaptability to harsh mechanical microenvironments [120,121]. Such “mechanically empowerment” CAR-T cells demonstrate enhanced tumor infiltration, prolonged persistence, and superior anti-tumor activity upon reinfusion, offering a novel strategy to improve adoptive cell therapy [[122], [123], [124]]. Therefore, mechanical pre-conditioning represents an important approach for optimizing immune cell function.

#### 2.2.2 Mechanical training and sustained polarization in macrophages and myeloid cells

Myeloid cells, particularly macrophages, also possess mechano-memory capabilities, a process termed “mechanical training”. Exposure to high-stiffness ECM can induce a long-lasting tumor-promoting (M2-like) phenotype sustained through YAP activation and metabolic reprogramming [89]. Studies show that a brief stiffness stimulus triggers lasting changes in macrophage mitochondrial metabolism, including enhanced glycolysis and reduced oxidative phosphorylation-a state termed “mechanotolerance” [19,125]. This metabolic state is “fixed” via a YAP-dependent transcriptional program, such that even upon transfer to a soft matrix, M2-type gene expression and immunosuppressive function are retained [19]. This mechanical training allows an initial mechanical stimulus to leave a lasting immunosuppressive imprint on the TME, which may persist even after primary tumor resection or shrinkage, potentially priming the soil for recurrence.

#### 2.2.3 Epigenetic and metabolic foundations

The long-term maintenance of mechano-immune memory depends on the synergistic action of epigenetic remodeling and metabolic reprogramming, which together constitute the molecular basis for the entrenchment of mechanical signals within immune cells [113,126].

At the epigenetic level, mechanical signals induce persistent changes in gene expression by altering chromatin state. Studies show that a stiff matrix drives the integrin-YAP signaling axis to recruit histone-modifying enzymes to specific gene loci, causing promoter regions of stemness-related genes (e.g., TCF7) to acquire active histone marks (e.g., H3K4me3), maintaining an open chromatin state and promoting the generation of stem-like memory T cells [68,127,128]. More directly, nuclear deformation can alter chromatin accessibility, and mechanical stress can drive assembly of stem cell gene super-enhancers by regulating phase separation of TAZ and NANOG [129].

At the metabolic level, mechanically guided reprogramming provides energy and molecular substrates for memory maintenance. Mechanically trained macrophages exhibit enhanced aerobic glycolysis; the metabolic byproduct lactate can inhibit histone deacetylase activity, leading to global histone hyperacetylation and consolidation of a pro-tumorigenic gene expression program [48]. Meanwhile, mechanical signals affect cellular metabolism by regulating mitochondrial dynamics. Increased mitochondrial fission is linked to T cell exhaustion, and changes in mitochondrial morphology directly affect levels of key metabolites like acetyl-CoA and α-ketoglutarate, thereby regulating histone acetylation and DNA methylation [[130], [131], [132]]. This forms a metabolic-epigenetic positive feedback loop that ensures long-term persistence of the cell state post-training [45]. Thus, epigenetic mechanisms provide heritable “software” encoding for mechanical memory, while metabolic reprogramming supplies the “hardware” support for its maintenance.

### 2.3 Pillar III: plasticity of the mechano-immunological landscape

The MIL is not a static background but exhibits high spatiotemporal heterogeneity and plasticity. This plasticity stems from the non-uniform distribution of mechanical signals across tumor regions and their dynamic crosstalk with complex biochemical networks.

#### 2.3.1 Mechanical heterogeneity in tumor regions shapes the spatial distribution of immune cells

**Fig. 2:**
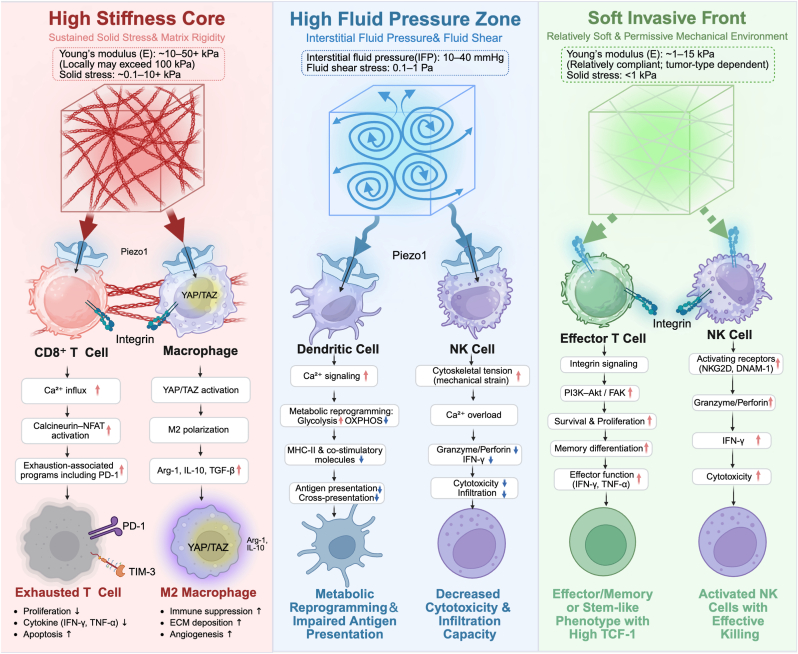
**Mechanoimmunological Landscape: Mechanical Heterogeneity Spatially Orchestrates Diverse Immune Cell Fates within the Tumor Microenvironment.** This figure illustrates how TME mechanical heterogeneity spatially encodes immune cell states, with three biophysically distinct zones: (1) High stiffness core (E = 10–50 kPa, solid stress 0.1–10 kPa): Chronic Piezo1/integrin signaling drives CD8^+^ T cell exhaustion (PD-1/TIM-3) via Ca^2+^-calcineurin-NFAT, and M2 macrophage polarization via YAP/TAZ. (2) High fluid pressure zone (IFP = 10–40 mmHg, shear = 0.1–1 Pa): Piezo1 impairs DC antigen presentation (reduced MHC II) and NK cytotoxicity (decreased granzyme B) via Ca^2+^ overload. (3) Soft invasive front (E = 1–15 kPa, solid stress<1 kPa): Integrin-PI3K/AKT/FAK signaling promotes stem-like TCF-1^+^ effector T cells and NK killing via NKG2D/DNAM-1. This map reveals mechanical geography as an immune fate determinant, offering a spatial blueprint for targeting physical immunosuppression. Piezo1, Piezo-type mechanosensitive ion channel component 1; CD8^+^ T Cell, Cluster of Differentiation 8 positive T Cell; Ca^2+^, Calcium ion; PD-1, Programmed Cell Death Protein 1; TIM-3, T-cell Immunoglobulin and Mucin-domain containing-3; Arg-1, Arginase-1; IL-10, Interleukin-10; M2 Macrophage, M2 type Macrophage; YAP, Yes-associated protein; TAZ, Transcriptional coactivator with PDZ-binding motif; NK Cell, Natural Killer Cell; TCF-1, T-cell Factor 1. This figure was created using BioRender (https://biorender.com/).

Significant mechanical heterogeneity exists within tumors. Typically, the tumor core, due to excessive ECM deposition, collagen cross-linking, and solid stress accumulation, exhibits markedly increased matrix stiffness and compressive stress [3]. These high-stiffness regions often accumulate functionally exhausted CD8^+^ T cells and M2-type tumor-associated macrophages, displaying an immune-excluded or immunosuppressive phenotype [89,133,134], which is often described as an “immune desert” or immune-excluded “cold” tumor structure (Fig. 2). Recent studies have further confirmed that tumor cell-derived collagen and other ECM components can form physical barriers, which directly mediate immune cell exclusion and lead to acquired resistance to immune checkpoint inhibitors, providing direct evidence for mechanical barrier-driven immunosuppression in non-small cell lung cancer and other solid tumors [135]. Conversely, at the tumor invasive front or metastatic sites, the ECM is relatively softer, and fluid shear stress may be higher [3,22]. This mechanical environment is generally more permissive for the infiltration and functional activation of effector T cells and NK cells [136,137].

This mechanically guided immune zonation is a result of tumor immunoediting and directly impacts immunotherapy efficacy. For example, treatment strategies targeting the stiff core (e.g., LOX inhibitor-mediated de-stiffening) may need to be combined with immune-activating strategies at the invasive front to achieve comprehensive spatial immune control [133,138,139].

#### 2.3.2 Crosstalk between mechanical and biochemical signals (e.g., cytokines)

Within the MIL, mechanical and biochemical signals do not operate independently but engage in complex crosstalk to co-regulate immune outcomes.

On one hand, biochemical signals can modulate cellular sensitivity to mechanical cues. For instance, TGF-β signaling can upregulate integrin expression and enhance cytoskeletal contractility, thereby amplifying cellular response to matrix stiffness and synergistically promoting fibroblast activation and further ECM stiffening [140,141]. On the other hand, immune signals can reciprocally regulate mechanical properties. IFN-γ secreted by activated T cells can alter macrophage cytoskeletal organization and mechanics, affecting their migration and phagocytic capacity [142,143].

Notably, some oncogenic driver mutations (e.g., in KRAS, PIK3CA, or TP53 deletion) can reshape cellular mechanosensory networks, altering their response patterns to ECM tension and stiffness, thereby sustaining YAP/TAZ signaling in high-pressure microenvironments [96,144]. For example, cells carrying oncogenic KRAS mutations exhibit fundamentally altered responses to mechanical stimuli (e.g., gut peristalsis), with a significantly lowered activation threshold for mechanosensitive pathways like YAP [144,145]. This synergy and antagonism between mechanical and chemical signals collectively shape an intricate immune-regulatory network, enabling the MIL to dynamically adapt and providing multiple potential targets for therapeutic intervention.

In summary, the plasticity of the MIL is multi-dimensional, spanning from matrix microstructure to cellular responses and from signaling pathway interplay to immune cell migration. Decoding this dynamic plasticity provides a foundation for future Mechano-immunological Landscape Remodeling Therapy (MILRT). The systematic elaboration of these three pillars—sensors (checkpoints), persistent imprints (memory), and dynamic adaptability (plasticity)—collectively argue for a therapeutic strategy that actively remodels, rather than merely navigates, the mechanical immune terrain.

## 3 A new paradigm for targeted mechano-immunotherapy: MILRT

Based on a systematic understanding of the Mechano-immunological Landscape (MIL), we propose a novel paradigm termed “Mechano-immunological Landscape Remodeling Therapy” (MILRT). This approach involves multidimensional interventions targeting both the tumor's mechanical ecology and its immune effector circuits, with the goal of reversing an immunosuppressive MIL into an immunosupportive one, thereby establishing a therapeutic positive feedback loop of perfusion, delivery, recognition, effector function, and memory [146,147](Fig. 4).

### 3.1 Strategy I: Tumor Mechanical microenvironment “de-stiffening”

The “de-stiffening” strategy aims to reduce the physical barriers posed by an aberrantly stiff extracellular matrix (ECM), thereby improving tissue perfusion, drug delivery, and immune cell infiltration [9], [148]. As previously discussed, drug repurposing represents a promising approach to tackle TME complexity, and de-stiffening is a direct application of this concept within the MILRT framework.

#### 3.1.1 LOX/LOXL2 inhibitors

Lysyl oxidase (LOX) and its isoforms are key enzymes that catalyze collagen cross-linking, directly driving ECM stiffening [149]. Inhibiting LOX/LOXL2 activity can effectively reduce matrix stiffness and enhance immune cell penetration [139]. Upregulation of LOXL2 expression is closely associated with matrix stiffening and promotes cancer cell invasion via activation of the mechanosensitive ion channel Piezo1 [150]. Preclinical studies show that LOX inhibitors can reverse fibrosis, enhance T cell infiltration into tumor cores, and synergize significantly with PD-1 blockade [53,54]. Thus, targeting LOX/LOXL2 is a foundational de-stiffening strategy.

#### 3.1.2 Angiotensin Receptor Blockers (e.g., losartan)

Angiotensin II receptor blockers (ARBs) such as Losartan possess anti-fibrotic properties by inhibiting the TGF-β pathway and reducing collagen synthesis and deposition [57,151]. In models of pancreatic and breast cancer, Losartan treatment reduces tumor stiffness, improves blood perfusion, and enhances the delivery of both chemotherapeutic agents and immune cells [57,58]. Studies on Losartan-based nanocomposite hydrogels demonstrate that reshaping the tumor mechanical microenvironment can mitigate delivery limitations and drug resistance, supporting its clinical translation for mechanical intervention [152].

#### 3.1.3 Hyaluronidase

Hyaluronic acid (HA) is a major glycosaminoglycan in the tumor ECM. Its abnormal accumulation increases interstitial fluid pressure (IFP), compresses vasculature, and hinders immune cell migration [59,153]. PEGylated human recombinant hyaluronidase (PEGPH20) degrades tumor-associated HA, effectively reducing IFP, improving vascular permeability and perfusion, and facilitating immune cell entry into the TME [60]. In PDAC models, PEGPH20 combined with chemotherapy improves drug delivery and survival, with early data suggesting it may help create a microenvironment more conducive to immunotherapy [59].

#### 3.1.4 Clinical translation of de-stiffening strategies

Beyond preclinical validation, several de-stiffening agents have advanced to clinical trials (Table 3). LOX/LOXL2 inhibitors: Simtuzumab (GS-6624), a humanized monoclonal antibody against LOXL2, was evaluated in a Phase II trial for PDAC (NCT01472198). Although the trial did not meet its primary endpoint of overall survival, post-hoc analysis suggested potential benefit in patients with high LOXL2 expression, and safety data showed manageable fatigue and edema [154]. A Phase I trial of the LOX inhibitor PXS-5505 in myelofibrosis (NCT04644029) is ongoing, with early reports indicating reduced collagen cross-linking and improved marrow fibrosis [155]. Losartan: A landmark Phase II trial (NCT01821729) in patients with resectable PDAC demonstrated that neoadjuvant losartan combined with FOLFIRINOX chemotherapy increased R0 resection rate and intratumoral CD8^+^ T cell infiltration, with no additional toxicities beyond chemotherapy [156]. A subsequent Phase III trial (NCT03563248) is currently evaluating losartan plus chemotherapy in locally advanced PDAC, with progression-free survival as the primary endpoint [157]. PEGPH20: The Phase III HALO-109-301 trial (NCT01959139) tested PEGPH20 plus nab-paclitaxel/gemcitabine in HA-high metastatic PDAC. While the overall survival benefit was not statistically significant, the HA-high subgroup showed improved median survival (11.5 months vs. 8.5 months), and adverse events included thromboembolic events and muscle spasms [158]. A Phase Ib/II trial of PEGPH20 combined with pembrolizumab (NCT02715804) revealed enhanced CD8^+^ T cell density in tumor biopsies, but Grade 3–4 thromboembolism occurred in 15% of patients, necessitating prophylactic anticoagulation [159]. These clinical data confirm that de-stiffening strategies are clinically feasible, with efficacy signals in selected patient populations, and highlight the need for biomarker-guided patient selection and optimized combination regimens to improve the therapeutic index.

In summary, de-stiffening strategies pharmacologically or enzymatically target key ECM components to reduce IFP, alleviate vascular compression, and enhance the delivery of therapeutic agents and immune cells. This is a critical first step in breaking down physical barriers and reversing immune exclusion [153], establishing a permissive foundation for subsequent immune cell engagement and checkpoint blockade within the multimodal MILRT framework [152](Table 1).

### 3.2 Strategy II: immune cell “mechanical empowerment”

In the stiff, compressed TME, immune cells face a “mechanical adaptation threshold” dictated by ECM structure and intrinsic stress gradients [146,160]. The efficacy of effector cells like T cells and NK cells depends not only on biochemical signals but also on their ability to maintain cytoskeletal integrity, sense mechanical cues, and sustain metabolic fitness under force [161,162]. Therefore, the goal of “mechanical empowerment” is to endow immune cells with a mechanically resilient phenotype prior to engagement in the TME.

#### 3.2.1 *In vitro* mechanical training

Conventional CAR-T or NK cell expansion systems often use soft matrices, leaving cells poorly adapted to the high-stiffness TME and prone to impaired infiltration, cytoskeletal dysfunction, and rapid exhaustion [134,163]. The concept of *in vitro* mechanical training leverages engineered biomaterial platforms, specifically stiffness-tunable 3D hydrogels, to precondition effector cells. By culturing CAR-T or NK cells within these synthetic or natural polymer-based matrices (which replicate the pathophysiological stiffness range of tumors, ∼5–30 kPa), their mechanical adaptability is proactively enhanced [61,62]. This biomaterial-driven priming actively reprograms the integrin-FAK-YAP axis, fortifies the cytoskeleton, and boosts metabolic resilience [68,164]. Mechanically primed CAR-T cells exhibit superior tumor infiltration, resistance to exhaustion, and persistent killing activity in vivo, highlighting the potential of mechanical preconditioning as a key module in next-generation cell therapy manufacturing [165].

#### 3.2.2 Nanomaterial regulation

Nanotechnology provides powerful biomaterial tools for in vivo mechanical empowerment [166]. Engineered nanoparticles function not only as conventional delivery vectors but also as direct “artificial mechanosensors” [152] or local mechanical micro-environment modulators [167]. By precisely tuning nanoparticle elasticity, topography, and surface mechanics, these biomaterials can be designed to activate specific mechanosensitive pathways (e.g., Piezo1) in immune cells, thereby reprogramming their function—such as driving macrophage polarization from M2 to M1 phenotype—to overcome physical immunosuppressive barriers (Fig. 1). Recent work in nanomechanical immune engineering shows that tuning nanoparticle elasticity can activate Piezo1 in macrophages, driving their polarization from a tumor-promoting M2 toward an antitumor M1 phenotype [63], exemplifying direct nanomaterial-mediated regulation of immune cell mechanosensing.

The mechanical empowerment strategy transforms passive cells into mechanically competent agents, aiming to endow them with the capacity to withstand and adapt to physical adversity. This represents a promising approach to improve adoptive cell therapy in solid tumors. This approach positions nanomaterials not merely as carriers, but as active pharmacologic agents capable of “prescribing” specific mechanical inputs to immune cells, offering a novel avenue for immuno-modulation rooted in materials science and drug delivery.

### 3.3 Strategy III: mechano-immunological checkpoint blockade

Mechano-immunological checkpoints are key nodes within the MIL that transduce physical cues into immune-regulatory signals. Their dysregulated activation drives mechanically induced immune suppression. Targeting these nodes can directly block this harmful signal transduction.

#### 3.3.1 Modulating PIEZO1

The mechanosensitive channel PIEZO1 exhibits a context-dependent, dual role in T cells: while moderate activation may support migration, chronic stimulation in a stiff TME leads to Ca^2+^-mediated functional exhaustion [17,168]. This establishes PIEZO1 as a bona fide mechano-immunological checkpoint [169] [16]. Therefore, precise pharmacological modulation using agonists such as Yoda1 or inhibitors such as GsMTx4 in a context-specific manner represents a promising therapeutic direction [64,65]. However, developing selective and safe modulators for ion channels like PIEZO1 presents distinct drug discovery challenges, necessitating innovative approaches in medicinal chemistry and targeted delivery to achieve tissue- and context-specific effects.

#### 3.3.2 YAP/TAZ-TEAD inhibitors

YAP/TAZ are core transcriptional effectors of mechanical signals, promoting tumor progression and immune suppression [170]. In myeloid cells, YAP/TAZ activation enhances immunosuppressive function [171,172]. Several small-molecule inhibitors disrupting the YAP/TAZ-TEAD interaction are in preclinical development [66,67]. Their ability to dually target tumor cells and immunosuppressive stromal populations makes them pivotal agents within the MILRT paradigm.

#### 3.3.3 Integrin function modulation

**Fig. 3:**
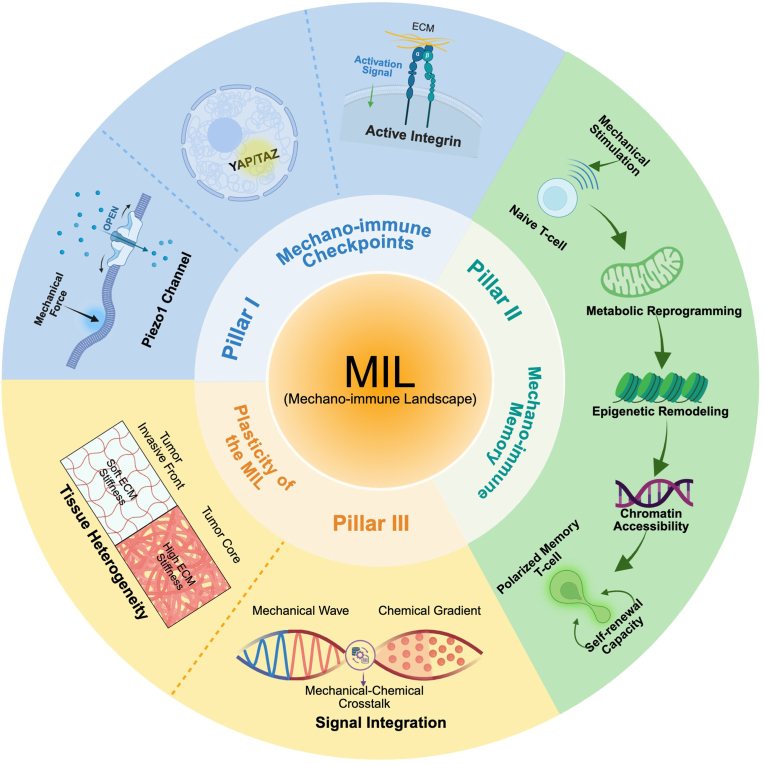
**Core Pillars of the Mechano-immunological Landscape (MIL) in the Tumor Microenvironment.** This schematic illustrates the three interconnected pillars underpinning the Mechano-immunological Landscape (MIL), a dynamic framework integrating mechanical cues with immune function within tumors. Pillar I (Mechano-immunological Checkpoints) depicts key molecular sensors such as the ion channel PIEZO1, integrin adhesion molecules, and the transcriptional co-activators YAP/TAZ that convert physical forces into immune-regulatory signals. Pillar II (Mechano-immune Memory) visualizes the long-term imprint of mechanical exposure on immune cells, showing how mechanical priming drives epigenetic and metabolic reprogramming to sustain stem-like or polarized states, such as in memory T cells. Pillar III (Landscape Plasticity) highlights the spatial heterogeneity and crosstalk within the TME, where mechanical gradients and chemical signals intersect to shape immune cell distribution and function. Together, these pillars form a cohesive and adaptable network, offering a foundational view for developing mechano-immunotherapeutic strategies aimed at remodeling the immunosuppressive tumor milieu. MIL, Mechano-immunological Landscape; ECM, Extracellular Matrix; YAP, Yes-associated protein; TAZ, Transcriptional coactivator with PDZ-binding motif; Piezo1, Piezo-type mechanosensitive ion channel component 1. This figure was created using BioRender (https://biorender.com/).

Integrins are primary sensors of ECM mechanics [13]. Mechanical signals through β2 integrins can decouple T cell proliferation from differentiation, promoting the generation of stem-like, persistent CAR-T cells [68]. This suggests that modulating integrin function via agonistic or blocking antibodies can precisely tune immune cell behavior in mechanical contexts (Fig. 3), for instance, to enhance infiltration or preserve memory phenotypes [69,83,173]. Thus, integrin modulation is a promising strategy for engineering next-generation cell therapies suited for mechanically challenging TMEs.

**Fig. 4:**
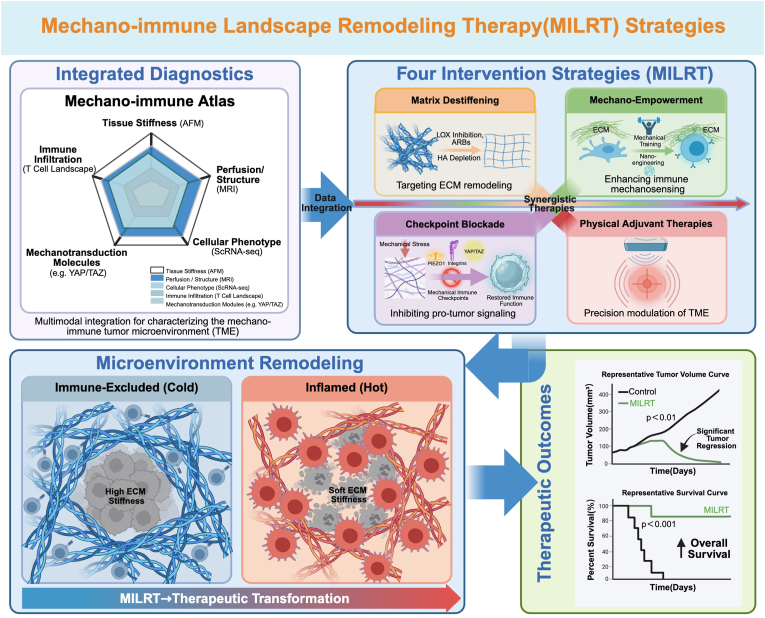
**Remodeling the Tumor Mechano-immune Niche: The MILRT Blueprint for Precision Immunotherapy.** This figure presents the conceptual pipeline of Mechano-immunological Landscape Remodeling Therapy (MILRT), outlining a systematic blueprint for precision immunotherapy that spans from integrated diagnostics to therapeutic transformation. The framework begins with constructing a multidimensional Integrated Mechano-immune Atlas, which synthesizes key parameters such as tissue stiffness (measured via AFM), vascular perfusion and architecture (via MRI), and high-resolution immune phenotypes (via scRNA-seq). Guided by this atlas, four synergistic Niche-Targeted Interventions are deployed (1) ECM Destiffening (e.g., using LOX inhibitors or ARBs): to reduce physical barriers (2) Mechano-checkpoint Blockade (e.g., targeting PIEZO1 or YAP/TAZ); to disrupt immunosuppressive signaling; (3) Mechanical Empowerment of immune effector cells to enhance their adaptability; and (4) Physical Adjuvant Therapies (e.g., ultrasound or radiotherapy) for localized modulation. Together, these strategies are designed to drive a Therapeutic Niche Transformation, conceptually converting an immune-excluded (“cold”), stiff tumor microenvironment into an inflamed (“hot”), immune-permissive niche. The expected therapeutic outcome of this integrated approach is illustrated by representative curves showing tumor volume reduction and a potential survival benefit, highlighting the promising rationale of MILRT as a transformative strategy to overcome resistance in solid tumor immunotherapy. MILRT, Mechano-immune Landscape Remodeling Therapy; AFM, Atomic Force Microscopy; MRI, Magnetic Resonance Imaging; scRNA-seq, single-cell RNA sequencing; ECM, Extracellular Matrix; LOX, Lysyl Oxidase; HA, Hyaluronic Acid; Piezo1, Piezo-type mechanosensitive ion channel component 1; YAP, Yes-associated protein; TAZ, Transcriptional coactivator with PDZ-binding motif; TME, Tumor Microenvironment. This figure was created using BioRender (https://biorender.com/).

### 3.4 Strategy IV: physical therapies as immune adjuvants

Physical therapies utilize non-pharmaceutical energy to directly remodel the MIL's physical properties and induce immunogenic effects, representing a unique component of MILRT.

#### 3.4.1 Ultrasound

Low-intensity pulsed ultrasound exerts mechanical stress via acoustic radiation force and cavitation, inducing immunogenic cell death and antigen release while improving vascular function and reducing IFP to promote immune cell infiltration [70,174]. Focused ultrasound combined with immune checkpoint inhibitors has shown preliminary synergistic efficacy in clinical exploration.

#### 3.4.2 Radiotherapy

Beyond direct cytotoxicity, radiotherapy damages the ECM, reduces local stiffness, and induces tumor neoantigen release, thereby reshaping the local immune landscape and converting “cold” tumors to “hot” [71,175]. This radiosensitizing effect on the immune microenvironment is well-documented, as in the synergy between radiotherapy and pembrolizumab in the treatment of non-small cell lung cancer [72,176].

#### 3.4.3 Photothermal/electro-stimulation

Photothermal therapy generates localized hyperthermia for ablation while inducing immunogenic death via thermal stress [73]. Electrical stimulation modulates cell membrane potential and ion channels to influence cell mechanosensing and migration [74,177]. Research has created mechanotransduction-guided vaccines that use physical stimulation to spatiotemporally regulate anti-tumor immunity, demonstrating the potent synergy between physical therapy and immunological engineering [75].

As immune adjuvants, physical therapies offer advantages such as non-invasiveness, local focality, and controllable side effects, enabling rapid and direct modification of the TME to favor immune activity (Table 1).

In conclusion, the MILRT paradigm transcends the biochemical focus of traditional immunotherapy by integrating the physical properties of tumors with immune responses into a unified framework. The four interlocking strategies of de-stiffening, mechanical empowerment, checkpoint blockade, and physical adjuvant therapy offer a multi-pronged approach to remodel the immunosuppressive TME (Fig. 1). As our understanding of mechano-immunology deepens and bioengineering tools advance, MILRT is poised to evolve from a conceptual framework into a practical cornerstone of next-generation solid tumor immunotherapy.

### 3.5 Synergistic integration of MILRT strategies

The synergistic logic of MILRT combinatorial strategies is underpinned by mechanistic interdependence among the four pillars. For instance, de-stiffening (Strategy I) combined with immune cell mechanical empowerment (Strategy II) addresses both the physical barrier and the effector cells’ intrinsic capacity to navigate residual forces. Preclinically, treatment with the matrix-modifying agent losartan (which reduces collagen cross-linking and stiffness) followed by adoptive transfer of mechano-engineered T cells expressing constitutively active Piezo1 has been shown to increase tumor infiltration by 3.5-fold and improve overall survival in a murine breast cancer model [178]. Similarly, combining de-stiffening with mechano-immunological checkpoint blockade (Strategy III), for example using a TGF-β receptor inhibitor to soften the stroma alongside a YAP/TEAD inhibitor to block mechanically induced PD-L1 upregulation, resulted in a 70% reduction in tumor volume and a 4-fold increase in CD8^+^ T cell effector function compared to monotherapy in a pancreatic ductal adenocarcinoma model [179]. Furthermore, the integration of physical therapies (Strategy IV) such as focused ultrasound-mediated mechanical priming with checkpoint blockade has demonstrated enhanced intratumoral T cell infiltration (2.8-fold) and durable memory responses in a syngeneic melanoma model, attributed to transient matrix softening and increased antigen release [180]. A triple combination of stromal de-stiffening, YAP/TAZ inhibition, and anti-PD-1 therapy in a stiff matrix-associated lung cancer model achieved complete tumor regression in 40% of mice, with significant upregulation of pro-inflammatory cytokines (IFN-γ, TNF-α) and downregulation of exhaustion markers (PD-1, TIM-3) [181]. These preclinical data collectively validate the concept that MILRT strategies function synergistically by sequentially dismantling mechanical barriers, empowering effector cells, blocking mechano-immune checkpoints, and providing localized physical stimulation, thereby converting the immunosuppressive MIL into a permissive milieu for durable antitumor immunity [182]. Future studies should systematically optimize the timing, dose, and sequence of these modalities to maximize therapeutic synergy while minimizing toxicity.

### 3.6 Potential resistance mechanisms and countermeasures to MILRT

Despite the conceptual promise of MILRT, clinical translation will likely encounter acquired resistance, analogous to that observed with conventional immunotherapies. Understanding these resistance mechanisms is essential for designing durable therapeutic strategies.

#### 3.6.1 Compensatory mechanical signaling pathways

A major resistance mechanism involves the activation of compensatory mechanotransduction pathways. For instance, sustained inhibition of LOX/LOXL2 (Strategy I) may upregulate alternative cross-linking enzymes such as transglutaminase-2 or lysyl oxidase-like 3, leading to ECM re-stiffening [183]. Similarly, long-term PYK2 blockade could induce compensatory activation of FAK or Src kinases, re-establishing mechano-immunosuppressive signaling [184]. Countermeasure: Combinatorial targeting of parallel mechanosensors (e.g., dual inhibition of PYK2 and FAK) or intermittent dosing schedules to prevent adaptive rewiring [185].

#### 3.6.2 Immune cell mechanical exhaustion and epigenetic stabilization

Mechanical empowerment (Strategy II) may initially enhance effector function, but chronic exposure to residual stiff matrix could drive epigenetic reprogramming that locks T cells into an exhausted state, even if the mechanical milieu is partially corrected. For example, sustained OSR2 upregulation in a stiff microenvironment induces heritable chromatin modifications that persist after de-stiffening [17]. This “mechanical memory” limits the durability of empowerment. Countermeasure: Combining mechanical empowerment with epigenetic modifiers (e.g., G9a inhibitors) to erase exhaustion-associated marks, or cyclic mechanical conditioning to prevent epigenetic fixation [186].

#### 3.6.3 Stromal reorganization and fibroblast plasticity

De-stiffening strategies (Strategy I) can trigger a reactive stromal response. Cancer-associated fibroblasts may shift from a matrix-depositing phenotype to a pro-inflammatory phenotype that paradoxically recruits myeloid-derived suppressor cells (MDSCs) [187]. In preclinical models, losartan-induced matrix softening was followed by a transient wave of MDSC infiltration, reducing the benefit of subsequent checkpoint inhibition [188]. Countermeasure: Timing the administration of de-stiffening agents to avoid a permissive window for MDSC recruitment, or co-targeting MDSC chemotaxis (e.g., CXCR2 inhibitors) [189].

#### 3.6.4 Checkpoint feedback and upregulation of alternative checkpoints

Mechano-immunological checkpoint blockade (Strategy III) may trigger feedback upregulation of other inhibitory receptors. For example, YAP/TEAD inhibition to downregulate PD-L1 can lead to compensatory upregulation of TIM-3 and LAG-3, driven by residual mechanical stress [190]. Similarly, PYK2 inhibition has been reported to increase expression of the checkpoint molecule VISTA on myeloid cells [191]. Countermeasure: Multi-checkpoint blockade (e.g., combining YAP/TEAD inhibitors with anti-TIM-3 antibodies) or dynamic monitoring of checkpoint expression to adjust combination strategies [192].

#### 3.6.5 Physical therapy resistance and immunogenic tolerance

Repeated application of physical therapies (Strategy IV) such as focused ultrasound or radiotherapy may induce immunogenic tolerance. For instance, high-intensity focused ultrasound can promote HSP70 release initially, but repeated treatment may exhaust dendritic cell cross-presentation capacity or induce T cell anergy [193]. Countermeasure: Optimizing pulse parameters and fractionation schedules, and combining physical therapies with dendritic cell-boosting adjuvants (e.g., TLR agonists) [194].

MILRT resistance is likely to be multi-faceted, involving mechanical, epigenetic, stromal, and checkpoint-related adaptations. Pre-emptive combination strategies, biomarker-guided adaptive regimens, and intermittent dosing protocols will be critical to overcome these resistance mechanisms and realize the full potential of mechano-immunotherapy.

In conclusion, the MILRT paradigm transcends the biochemical focus of traditional immunotherapy by integrating the physical properties of tumors with immune responses into a unified framework. The four interlocking strategies are designed to operate synergistically: de-stiffening (Strategy I) breaks down physical barriers to access; mechanical empowerment (Strategy II) prepares immune effectors to thrive in the remodeled but still challenging niche; mechano-checkpoint blockade (Strategy III) interrupts the deleterious signaling born of mechanical stress; and physical adjuvants (Strategy IV) provide localized, on-demand modulation of the MIL (Fig. 1). This multi-pronged approach aims to convert an immunosuppressive “cold” tumor MIL into an immunosupportive “hot” one. As our understanding of mechano-immunology deepens and bioengineering tools advance, MILRT is poised to evolve from a conceptual framework into a practical cornerstone of next-generation solid tumor immunotherapy.

## 4 Challenges and future perspectives

**Fig. 5:**
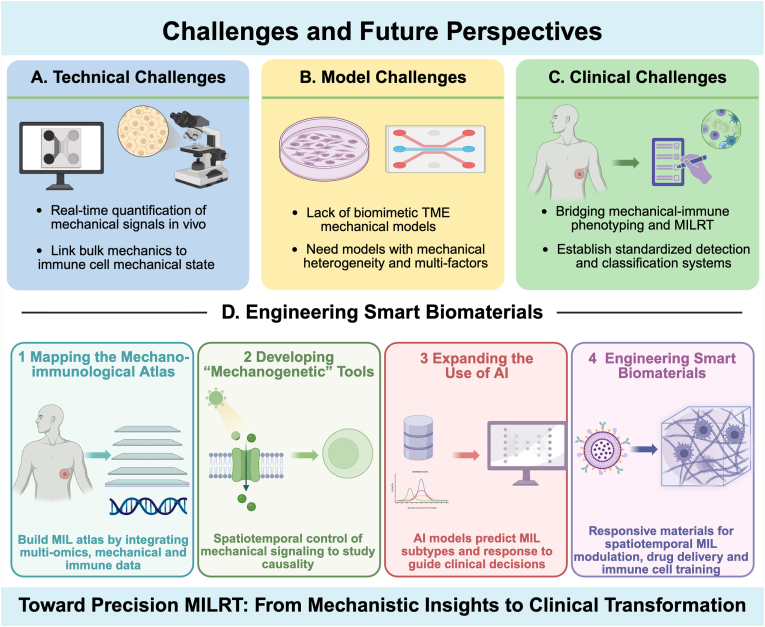
**The Mechano-immunological Landscape (MIL): Challenges and Future Perspectives** This figure outlines key challenges and future directions in the mechano-immunological landscape (MIL). The top panel highlights three critical challenges: (1) Technical Challenges (real-time in vivo mechanical signal quantification and linking bulk mechanics to immune cell mechanical states), (2) Model Challenges (lack of biomimetic tumor microenvironment (TME) mechanical models and need for heterogeneous, multi-factor models), and (3) Clinical Challenges (bridging mechanical-immune phenotyping to mechano-immunological therapy (MILRT) and standardizing detection systems). The bottom panel presents four future strategies: (1) mapping the MIL atlas via multi-omics integration, (2) developing “mechanogenetic” tools for spatiotemporal control of mechanical signaling, (3) expanding AI applications to predict MIL subtypes and guide clinical decisions, and (4) engineering smart biomaterials for responsive MIL modulation, drug delivery, and immune cell training. The overall goal is to advance precision MILRT from mechanistic insights to clinical transformation. MILRT, Mechano-immune Landscape Remodeling Therapy; TME, Tumor Microenvironment. This figure was created using BioRender (https://biorender.com/).

Although the concept of the Mechano-immunological Landscape (MIL) provides a powerful theoretical framework for understanding tumor immunity and the proposed Mechano-immunological Landscape Remodeling Therapy (MILRT) shows considerable therapeutic potential, this emerging field still faces substantial challenges from basic research to clinical translation. Progress is currently hindered by three core issues; addressing these will be pivotal for future advancement (Fig. 5).

### 4.1 Technical Challenges: real-time sensing and quantification of mechanical signals in vivo

A major bottleneck in decoding the MIL is the real-time, dynamic, and high-resolution measurement of mechanical properties within living tumors and of the mechanical state of immune cells. While techniques like atomic force microscopy (AFM) offer high accuracy at the cellular or tissue level, they are largely restricted to *in vitro* or ex vivo samples and cannot capture the complex mechanical heterogeneity and dynamics of the tumor-host system in vivo [195,196].

In vivo mechanical imaging techniques, such as ultrasound elastography and magnetic resonance elastography (MRE), allow non-invasive assessment of whole-organ or whole-tumor stiffness [[197], [198], [199]]. However, their spatial resolution remains at the tissue scale, limiting analysis of intratumoral mechanical heterogeneity [200], and they cannot link bulk mechanical properties to the mechanical state (e.g., membrane tension, nuclear deformation) of specific immune cells [198,201,202].

Future advances will likely rely on multimodal integration. Novel mechanical probes compatible with live-cell microscopy, or Förster resonance energy transfer (FRET)-based tension biosensors, may enable real-time visualization of mechanical states in specific cellular subpopulations using multiphoton microscopy [201,203,204]. Furthermore, applying artificial intelligence (AI) to high-dimensional image data could help extract “mechanoradiomic” features from conventional medical images (e.g., CT, MRI), offering a feasible non-invasive approach for indirect assessment of the tumor MIL [205].

### 4.2 Model Challenges: the shortage of biomimetic TME mechanical models

A critical issue is whether *in vitro* models can faithfully recapitulate the mechanical heterogeneity of the human TME. Widely used 2D culture systems fail to mimic the complex 3D architecture and mechanical diversity of the in vivo TME [206]. Although 3D hydrogels and organoids represent an advance, most hydrogel systems exhibit uniform mechanical properties and cannot replicate the physiological mechanical gradients (from soft invasive fronts to stiff cores) or multidimensional factors like solid stress and interstitial fluid pressure [207,208].

Future work must focus on developing next-generation biomimetic models underpinned by advanced biomaterials to faithfully recapitulate the mechanical heterogeneity of the human TME. This requires the innovation of dynamic hydrogels with spatially graded stiffness, stress-responsive polymers, and organ-on-a-chip scaffolds that integrate controllable mechanical cues (e.g., cyclic stress, fluid shear). Such material-driven models are essential for multiscale analysis of mechano-immune crosstalk and for serving as high-fidelity platforms to test MILRT strategies. Patient-derived organoids offer a promising platform: by tuning matrix stiffness, ligand density, and 3D architecture, patient-specific “mini-tumors” with physiologically relevant mechanics can be constructed [209]. More advanced organ-on-a-chip systems can further integrate multiple components. For instance, fabricating regions of differing stiffness to mimic primary and metastatic sites, and co-culturing tumor, immune, and endothelial cells under controlled mechanical stimuli (e.g., cyclic pressure, fluid shear stress) [210,211]. Such *in vitro* personalized models that capture mechano-immune crosstalk will not only aid mechanistic studies but also serve as high-throughput platforms for testing MILRT strategies [212].

### 4.3 Clinical Challenges: bridging mechanical immune phenotyping and individualized MILRT

A central translational challenge is how to perform effective mechano-immunological phenotyping of patients. This requires moving beyond conventional genomic and pathological diagnostics to systematically integrate tumor physical properties (e.g., overall stiffness, stress distribution) with immune features (e.g., expression of mechano-immunological checkpoints, T cell mechanical memory). The goal is to establish a clinically applicable classification system to guide MILRT strategy selection [5,213].

Currently, clinical diagnostics rarely consider tumor physical properties or their immunomodulatory roles. Standardized protocols for acquiring, quantifying, and integrating multidimensional mechanical and immune data are lacking [1]. To address this, two key tasks are needed: 1. Standardized Detection Methods: Developing clinically feasible techniques to quantify tumor mechanical parameters (e.g., stiffness, solid stress) and key mechano-immunological checkpoint expression/activity (e.g., PIEZO1, YAP) [5,214]. 2. Clear Classification Criteria: Establishing a multidisciplinary framework involving radiology, pathology, biomechanics, and immunology experts to define mechano-immunological subtypes.

For example, combining non-invasive imaging, AFM on biopsies, and single-cell multi-omics could help classify patients into subtypes such as “fibrotic-immune excluded” or “mechanically heterogeneous-immune dysregulated” [195]. This would support tailored treatment selection (e.g., Losartan, PIEZO1 modulators, or mechanically-primed CAR-T cells) [213,215]. Only with such a comprehensive phenotyping system can MILRT advance toward individualized precision medicine.

### 4.4 Future directions

To overcome these challenges, future research should prioritize the following strategic directions [216].

1. **Mapping the Mechano-immunological Atlas**: Inspired by projects like The Cancer Genome Atlas, a large-scale interdisciplinary effort should systematically integrate patient multi-omics data, mechanical parameters, and immune cell infiltration patterns to construct MIL characteristic maps across tumor types and stages [[217], [218], [219]]. A comprehensive MIL database will facilitate identification of key driver modules, novel biomarkers, and therapeutic targets. Mechano-immunophenotyping techniques have certain clinical feasibility, but they face major challenges. Despite the conceptual appeal, translating mechano-immunophenotyping into routine clinical practice faces significant challenges. Current techniques for measuring tumor mechanical properties, such as ultrasound elastography, magnetic resonance elastography (MRE), and atomic force microscopy (AFM) on biopsies, each have inherent limitations. Ultrasound elastography and MRE provide non-invasive stiffness estimates but suffer from limited spatial resolution (typically >1 mm) and operator-dependent variability, making them unsuitable for characterizing intratumoral heterogeneity at the immune-cell scale [220,221]. Furthermore, these modalities measure bulk stiffness rather than the micro-mechanical environment that directly influences immune cell behavior. AFM offers nanoscale resolution but requires fresh tissue biopsies, which are invasive and subject to sampling bias [222]. In addition, standardizing mechanical measurements across institutions and equipment remains unresolved, and no universal consensus exists for reporting stiffness values (e.g., kPa vs. Young's modulus vs. shear wave velocity) in the context of immune infiltration [223]. To address these barriers, several practical optimization strategies are emerging. First, multimodal imaging fusion combining elastography with diffusion-weighted MRI or contrast-enhanced CT can provide complementary information on stiffness, perfusion, and vessel compression, enabling a more holistic assessment of the mechanical microenvironment [224]. Second, machine learning algorithms trained on large-scale elastography datasets are being developed to automatically segment tumor regions and predict immune infiltration patterns from stiffness maps, reducing operator dependency [225]. Third, liquid-based mechanical biomarkers, such as circulating tumor cell deformability and exosomal stiffness measured by microfluidics, offer a less invasive window into tumor mechanical properties and have shown early correlations with immunotherapy response [226]. Fourth, standardization efforts led by the quantitative imaging biomarkers alliance are establishing protocols for elastography quality assurance and inter-scanner comparability. Finally, organoid-based mechanical assays using patient-derived tumor organoids can recapitulate the native stiffness landscape *in vitro* and predict individual patient responses to de-stiffening agents [227]. These advances, while still in early stages, suggest that with continued technical refinement and multi-institutional validation, mechano-immunophenotyping can ultimately become a clinically feasible tool for guiding personalized MILRT strategies.
2. **Developing “Mechanogenetic” Tools**: To establish causality between mechanical signals and immune phenotypes, we need tools for spatiotemporally precise control of mechanical signaling in living cells. “Mechanogenetics”, inspired by optogenetics, uses light- or magnetic-controlled protein components such as photosensitive mechanosensitive ion channels to activate or inhibit specific mechanical pathways in specific cells at specific times [[228], [229], [230]]. For instance, optogenetic Piezo1 tools enable precise study of T cell functional changes upon mechanical activation within defined tumor regions [228,231].
3. **Expanding the Use of Artificial Intelligence**: The complexity of the MIL exceeds conventional analytical capacity. AI, particularly machine learning, will be essential for interpreting such complex systems. By training AI models on integrated clinical, imaging, mechanical, and multi-omics data, we can build predictive algorithms to determine a tumor's MIL subtype and forecast its response to different MILRT strategies (e.g., whether to apply de-stiffening before immunotherapy) [[232], [233], [234], [235]].
4. **Engineering Smart and Responsive Biomaterials for Spatiotemporal MIL Modulation:** The clinical translation of MILRT will heavily rely on the development of next-generation biomaterials. Future research must focus on designing intelligent, stimuli-responsive materials capable of sensing and dynamically modulating the TME [216]. These include: (a) Degradable or mechanically adaptive matrices that respond to TME cues (e.g., MMPs, pH) to locally alleviate stiffness and release drugs; (b) Nanoparticle systems whose elasticity, adhesion, or drug release profiles are triggered by mechanical forces or TME biochemistry; and (c) 3D scaffolds and hydrogels with spatially patterned mechanical and biochemical cues hold great promise for predictive ex vivo testing and immune cell training. The integration of such biomaterials with drug delivery and cell engineering platforms will enable precise, feedback-controlled remodeling of the MIL, moving beyond static interventions towards adaptive and personalized mechano-immunotherapies. However, the clinical translation of *in vitro* mechanical training for immune cells-particularly for CAR-T and CAR-NK cells — faces several critical technical hurdles. First, scalable production: Current 3D hydrogel systems (e.g., collagen-alginate or PEG-based matrices) are often fabricated using manual or small-batch microfluidic methods, which yield limited cell numbers (typically <10^8^ cells per batch) and are not compatible with Good Manufacturing Practice (GMP) requirements [236]. Recent advances in automated bioreactor-based hydrogel crosslinking (e.g., using continuous flow photo-crosslinking) have demonstrated production of up to 10^11^ mechano-trained CAR-T cells per run while maintaining >90% viability, but these systems are not yet commercially validated [237]. Second, mechanical memory retention in vivo: T cells or NK cells trained in stiff or soft hydrogels undergo cytoskeletal remodeling and epigenetic changes that may revert after removal from the matrix and infusion into theTME. Studies show that the enhanced effector function and resistance to exhaustion elicited by 10 kPa hydrogels decays within 5–7 days after adoptive transfer, due to loss of FAK activation and re-expression of exhaustion-associated transcription factors [238]. Third, batch-to-batch consistency: Hydrogel stiffness, pore size, and ligand density are highly sensitive to fabrication conditions (temperature, pH, crosslinking time). Variability in these parameters leads to heterogeneous training outcomes, with reports of 30–50% coefficient of variation in cell activation markers across batches [239]. Advanced quality control tools, such as real-time fluorescence-based crosslinking monitoring and machine learning-assisted optimization of gel composition, have reduced batch variability to <10% in pilot studies [240]. Fourth, cell recovery without damage: Harvesting trained cells from 3D scaffolds without compromising viability or function remains challenging. Enzymatic digestion (e.g., collagenase) can cleave surface receptors, reducing CAR-T cell potency by up to 40% [241]. Bioengineered thermoresponsive hydrogels (e.g., poly(N-isopropylacrylamide)) that dissolve at physiological temperature without enzymes have been developed, achieving >85% viable cell recovery with retained mechano-memory [242]. These innovations, while still in preclinical stages, suggest that scalable GMP-compliant manufacturing protocols, combined with in vivo maintenance strategies and robust quality assurance, can overcome the current barriers and facilitate clinical translation of mechanical empowerment for adoptive cell therapies.

Such tools will optimize clinical trial design and serve as clinical decision-support systems for personalized mechano-immunotherapy.

## 5 Concluding remarks

The proposal of the Mechano-immunological Landscape (MIL) concept marks a profound paradigm shift in our understanding of tumor immunity, shifting from a predominantly “two-dimensional” focus on biochemical signaling networks toward a new stage of systematic cognition that incorporates mechanical, three-dimensional, and dynamic dimensions.

Under this new paradigm, the physical characteristics of tumors (stiffness, solid stress, fluid pressure) are no longer incidental background factors but core elements that actively shape immune responses and drive immunoediting. The MIL framework integratively links tumor physics with immune function, revealing mechanical signals as a core regulator of tumor-immune interactions.

Based on this understanding, we propose the emerging therapeutic paradigm of Mechano-immunological Landscape Remodeling Therapy (MILRT). MILRT transcends traditional immunotherapy focused on blocking or activating biochemical signals. It advocates a multi-target; multimodal combinatorial strategy aimed at reversing immunosuppression at its physical root. Its core logic is to dismantle physical barriers via de-stiffening, enhance immune cell efficacy via mechanical empowerment, reverse harmful signaling via mechano-immunological checkpoint blockade, and supplement with physical therapies to directly modulate the MIL. This seeks to convert an immunosuppressive “cold” tumor MIL into an immunosupportive “hot” one. For example, combining Losartan to reduce stiffness and improve T cell infiltration, with *in vitro* mechanical empowerment of CAR-T cells, and YAP/TAZ inhibitors to block mechano-transcriptional output, offers a promising synergistic strategy to overcome core dilemmas of immunotherapy resistance in solid tumors [152,243].

Looking ahead, research in the MIL field will deepen and expand. We anticipate that “mechanobiopsy” and “mechano-immunological phenotyping” will become integral to diagnostic and therapeutic planning. With the maturation of mechanogenetic tools and AI-driven predictive models, individualized MILRT will transition from concept to clinical reality. Ultimately, through continuous technological breakthroughs and deep interdisciplinary integration, the diagnosis and treatment targeting the MIL will become a highly dynamic frontier in tumor immunology and pharmacology. In-depth exploration and precise intervention of the Mechano-immunological Landscape will not only enrich our fundamental understanding of how physical and chemical laws intertwine in biology but is also expected to bring new dawn for countless cancer patients, advancing the grand goal from “treating cancer” to “curing the patient”.

## Ethics statement

Not applicable.

## Funding

This work was financially supported by the High-Level Talent Introduction Funds from the First Hospital of 10.13039/100012899Lanzhou University.

## CRediT authorship contribution statement

**Wen Li:** Writing – original draft. **Yuan-Yuan Xin:** Writing – review & editing. **Ming-Zhu Jin:** Writing – review & editing. **Wei-Lin Jin:** Writing – review & editing.

## Declaration of competing interest

The authors declare that they have no known competing financial interests or personal relationships that could have appeared to influence the work reported in this paper.

## Data Availability

No data was used for the research described in the article.
